# Apatinib Mesylate Inhibits the Proliferation and Metastasis of Epithelioid Malignant Peritoneal Mesothelioma *In Vitro* and *In Vivo*

**DOI:** 10.3389/fonc.2020.585079

**Published:** 2020-12-07

**Authors:** Zhi-Ran Yang, Zhi-Gao Chen, Xue-Mei Du, Yan Li

**Affiliations:** ^1^Department of Peritoneal Cancer Surgery, Beijing Shijitan Hospital, Capital Medical University, Beijing, China; ^2^Department of Research, Thorgene Co., Ltd., Beijing, China; ^3^Department of Pathology, Beijing Shijitan Hospital, Capital Medical University, Beijing, China

**Keywords:** malignant peritoneal mesothelioma, patient-derived xenograft model, primary cell line, toxicity, apatinib

## Abstract

**Objective:**

Malignant peritoneal mesothelioma (MPM) is a rare malignancy with few effective molecular therapies. In this study, we evaluated the anti-tumor activity and safety of apatinib, a vascular endothelial growth factor receptor 2 inhibitor, in MPM *in vitro* and *in vivo*.

**Methods:**

We established several patient-derived xenograft (PDX) models and primary cell lines of MPM. The cell lines were used to study the effects of apatinib on proliferation, cell cycle, migration, and apoptosis by CCK8, flow cytometry, wound-healing, Transwell, DAPI staining, and caspase-3 assays, respectively. For *in vivo* study, apatinib was delivered by gastric gavage into PDX models, and then efficacy and toxicity were determined by experimental peritoneal cancer index (ePCI) score and pathological examinations.

**Results:**

Our results showed that apatinib significantly inhibited the proliferation and migration of MPM cells *in vitro* and induced cell cycle arrest. Studies on PDX models concurred that apatinib effectively suppressed subphrenic and liver invasions of nude mice. Moreover, histopathological analysis found that lymphocyte infiltration, coagulation necrosis and eosinophilic cell fragments were detected in tumor tissues after apatinib treatment. Apatinib showed no obvious effects on body mass of models and did not affect function of important organs, except for occasional focal lymphoid infiltration of liver (16.7%) and cardiac muscle (16.7%).

**Conclusions:**

We successfully established MPM PDX models and primary cell lines, and confirmed that apatinib effectively inhibited proliferation and metastasis of MPM *in vitro* and *in vivo* study.

## Introduction

Malignant peritoneal mesothelioma (MPM) is a rare malignancy characterized by highly aggressive behavior and poor prognosis. MPM accounts for 10%–30% of all malignant mesothelioma, and the median survival time of patient limited 5 to 12 months (1, 2). To date, there is no effective targeted therapeutic approaches to MPM, and the Peritoneal Surface Oncology Group International (PSOGI) recommends the combination of cytoreductive surgery (CRS) with hyperthermic intraperitoneal chemotherapy (HIPEC) as the standard treatment (3, 4). The median survival of treated patients can be extended to 3 years, but some patients still showed no benefits from CRS + HIPEC (5–7). Therefore, there is an urgent need to explore new treatments.

Apatinib is a small molecule inhibitor that selectively targets the ATP binding site of the receptor tyrosine kinase vascular endothelial growth factor receptor-2 (VEGFR-2). Apatinib blocks the downstream signal transduction of VEGF pathway to inhibit neovascularization. Its clinical application has been approved for patients with advanced gastric cancer or gastric-esophageal junction cancer (8, 9). Apatinib has also showed anti-tumor effects on other kinds of tumors, such as sarcoma, breast cancer, ovarian cancer, and acute lymphoblastic leukemia (ALL) (10–14). Moreover, a recent case report provided supporting information for apatinib to treat epithelioid malignant plural mesothelioma (15). Nevertheless, its expected inhibitory effect on MPM remains to be elucidated.

In the current study, we applied MPM surgical specimens to establish patient-derived xenograft (PDX) models in nude mice, and to culture the primary cell lines. These models were used to evaluate the effects and toxicity of apatinib on MPM both *in vivo* and *in vitro*.

## Materials and Methods

### Patient and Tumor Sample

Tumor samples were obtained from a patient, who was diagnosed as epithelioid MPM. Resected tumors were used for the establishment of PDX models. The studies involving human participants were reviewed and approved by the Scientific Research Ethics Committee of Beijing Shijitan Hospital, Capital Medical University [Approval number: 2020 Research Ethics Review No. (2)]. Written informed consent was obtained from the individual for the publication of any potentially identifiable images or data included in this article.

### Reagents

Apatinib was provided by Hengrui Medicine Co., Ltd. (Jiangsu, China). For *in vitro* studies, apatinib was dissolved in 100% dimethyl sulfoxide (DMSO; Sigma, USA) to yield a 151 mM stock solution, which was then diluted to the specified concentration in subsequent experiment by using Dulbecco’s Modified Eagle’s Medium (DMEM; Thermo Fisher Scientific, USA). For *in vivo* studies, apatinib was diluted in 0.5% Carboxymethyl Cellulose-Na solution (CMC; Sigma, USA).

### Establishment of MPM PDX Model

#### Animals

Specific pathogen free BALB/c nu/nu mice, 4–5 weeks old, 16–18 g, were purchased from Beijing Vital River Laboratory Animal Technology Co., Ltd. (Beijing, China) and were raised individually in ventilated cages in a barrier environment at the temperature of 20°C–26°C, in the humidity of 40%–70% at Beijing Percans Oncology (Beijing, China). All experiments were performed under the guideline of the Declaration of Helsinki and Institutional Animal Care and Use Committee Health guidelines (IACUC20190306). And the animal study was reviewed and approved by the Scientific Research Ethics Committee of Beijing Shijitan Hospital, Capital Medical University.

#### Model Establishment

The method and process of establishing MPM models were described before (16). Briefly, Surgical specimens were inoculated subcutaneously on the back of nude mice using a 25G trocar sheath needle to develop the subcutaneous (s.c.) models, and when the volume of tumors reached 500 mm^3^, they were resected for histopathological characterization, establishment PDX models, and culture primary cells.

In terms of establishment PDX models, s.c. tumors were cut into pieces and homogenized in a glass tissue homogenizer containing 1.5 ml RPMI 1640 medium (Corning, New York, USA) to produce 2.5 ml tumor cell homogenate. Twenty-two nude mice were used for PDX models establishment and intervention study. Each nude mouse was inoculated with 100 μl of tumor cell homogenate in the left lower abdominal cavity. Mice was closely monitored daily, and the body weight was measured every three days.

### Establishment and Identification of the Primary Cell Lines

#### Isolation and Cell Culture

The s.c. tumors were transferred to a centrifuge tube containing Dispase® II (Sigma, USA) and digested on 37°C incubator for 1 h. And then the cell suspension was filtered using a 70 μm cell filter, and then centrifuged at 1600 r for 5 min, followed by re-suspending in culture medium.

The culture medium included DMEM containing 10% fetal bovine serum (FBS; Gibco, USA) and 100 U/ml penicillin and 100 μg/ml streptomycin (Gibco, USA), the cells were cultured at 37°C, 5% CO_2_ in a humidified Forma Steri-Cycle CO_2_ Incubator (Thermo Fisher Scientific, USA).

#### Swiss-Giemsa Staining

200 μl culture medium was added into each well of 24-well plate with Sterile round slides. Cell suspension of 3×10^4^ was added to each well. 24 h of incubation later, cells were fixed with ice-cold methanol for 3 min and then stained with Swiss-Giemsa (Solarbio, China) for 5 min. EVOS™ XL Core Configured Microscope (Thermo Fisher Scientific, USA) was used to observe the cell morphology.

#### Immunocytochemical Staining

Cells were added into 24-well plate according to method described above, and were fixed with ice-methanol for 20 min, blocked with 10% FBS for 30 min, and incubated with primary antibody at 37°C for 60 min, the primary antibodies were rabbit anti-human Calretinin antibody (Zhongshan Golden Bridge, Poly), mouse anti-human WT-1 antibody (Genetic technology, 6F-H2), mouse anti-human Cytokeratin 5/6 antibody (OriGene, OTI1C7), and mouse anti-human Ki-67 antibody (OriGene, UMAB107). Then the cells were incubated by using secondary antibody (Enzyme-labeled goat anti-mouse/rabbit IgG polymer; OriGene; USA) for 30 min. The signal of interest was captured using Zeiss Axio Scope.A1 (Carl Zeiss AG, Oberkochen, Germany).

### Cell Proliferation Assay

Cells were seeded onto 96-well plates at a density of 1×10^5^ cells/well in 100 μl of culture medium. Cells were treated with apatinib at different concentrations (0, 12.5, 25, 50, or 100 μM) for 24, 48, or 72 h. Each well was added with 10 μl CCK-8 and were then incubated for additional 2 h. The absorbance of the solution (OD_450_) was measured at the wavelength of 450 nm on a microplate reader (ELX800, Bio TEK, USA).

### Flow Cytometry

Cells were plated into six-well plates at a density of 3×10^6^ cells/well and treated with 50 μM apatinib for 24 and 48 h. Cells were the harvested and fixed with 70% ethanol at −20°C for overnight, and then were stained with 1 μg/ml propidium iodide (PI; Solarbio, Beijing, China) at 4°C for 30 min. The stained cells were analyzed by using flow cytometer (BD Accuri™ C6 Plus Flow Cytometer, Dickinson and Company BD Biosciences, USA). the results were analyzed by Modfit LT software.

### Cell Migration and Movement

#### Scratch Wound Healing Assay

Cells were seeded into the 6-well plates at density of 1×10^6^ cells/well. Assay was performed when the monolayer cells reached 100% confluence. Cell layer was wounded by scratching, and then cells were cultured in DMEM treated with apatinib (25 or 50 μM) or DMSO for 24 h as control. The wound was imaged under a microscope at 100× magnification 0 and 24 h after scratch, and the width was calculated by using Image J software. The following formula was used to analyze the biological effect of apatinib. Cell migration inhibition rate = (0 h scratch width-24 h scratch width)/0 h scratch width.

#### Transwell Migration Assay

Cells were harvested and re-suspended in serum-free DMEM at a density of 2×10^5^ cells/ml. 100 μl cells treated with apatinib (25 or 50 μM) were plated in the upper chamber (Corning Incorporated, Kennebunk, USA). 500 μl of DMEM containing 10% FBS were placed in the lower chambers. After incubation for 24 h, the cells on upper surface of the membrane were removed, and those on the bottom surface of membrane were fixed with ice-cold methanol for 5 min and stained with 0.1% crystal violet solution for 15 min. An invert microscope at 100× magnification was used to observe cell migration. The number of migrated cells was counted in five random fields. Image-pro plus was used to count the number of migrated cells.

### Apoptosis Assays

#### DAPI Fluorescence Staining to Detect Nucleus

Cells cultured on the round slides in 24-well plates were treated with or without 50 μM apatinib for 24 h, and were fixed with ice-methanol for 5 min, and then stained with DAPI for 5 min. The nucleus of stained cells was observed by using fluorescence microscopy (Olympus, BX43, Japan).

#### Caspase-3 Assays

Cell density was adjusted to 1×10^5^ cells/ml. Cell suspension was added to 96-well plate at 100 µL/well. Different concentrations of apatinib (1, 2, 4, 8, 16, 32, and 64 μM) were added and the incubated for 24 h. The activity of caspase-3 protein in the cells were measured by using caspase-glo^®^ 3/7 detection kit (Promega Corporation, Madison, USA). Luminescence value was read at the wavelength of 490 nm by using a microplate reader.

### Apatinib to Treat MPM PDX Model

Two weeks after grafting, two nude mice were randomly selected for immunohistochemistry (IHC) staining to confirm MPM. Eighteen nude mice were randomly divided into three groups: blank control, solvent control and apatinib groups (n=6 for each group). Blank control group received no intervention, solvent control group was administrated with 24 μg/g/day 0.5% CMC, and treatment group was treated with 100 μg/g/day apatinib delivered by intra-gastric gavage. The treatment process lasted for 2 weeks.

#### Gross Pathological Study

Mice were sacrificed for autopsy. Tumor growth and progression features were recorded. Experimental peritoneal cancer index (ePCI) was used to evaluate the extent of tumor dissemination, based on the published studies (16–18). The abdominal-pelvic cavity was divided into 4 subareas, and lesion size score (LS) in each subarea is determined by diameter of the largest tumor: LS-0, no visible tumor; LS-1, diameter ≤ 0.2 cm; LS-2, 0.2 cm < diameter ≤ 0.5 cm; LS-3, diameter > 0.5 cm; Malignant ascites, 1 point. The accumulative ePCI score ranges from 0 to 13.

#### Hematoxylin-Eosin and IHC Staining of Tissues

The method was performed as we described before (16), briefly, the tumors were fixed in 4% formaldehyde solution for 48 h, followed by routine dehydration, paraffin embedding and section. Hematoxylin-eosin (HE) staining was performed on Dako CoverStainer (Agilent Technologies, Inc., California, USA). IHC staining was performed on intelliPATH FLX (BIOCARE MEDICAL, LLC, California, USA) with Polymer Immunohistochemical Detection System (Wuxi OriGene Technologies, Inc., Wuxi, China). Primary antibodies, including anti-Calretinin, -Cytokeratin 5/6, -WT-1, -Ki-67 and -VEGFR-2 antibody (OriGene, Poly), were used for IHC analysis. Images were captured using Zeiss Axio Scope.A1. The positive rates of Ki-67 and VEGFR-2 were calculated by using Image-pro plus.

For toxicity evaluation, the organs of nude mice were harvested and fixed in 4% formaldehyde solution for 48 h. HE staining was performed according to the method described above. Blank control group was used as normal control, and abnormal histological signs under the microscope were considered as toxic reaction. All HE and IHC sections were reviewed by 3 senior pathologists who were blind to the design of the study.

### Statistical Analysis

Experiments were repeated at least three times. Data are presented in the form of mean ± standard deviation (SD) when normal distribution is satisfied. Statistical analyses and image processing were performed using GraphPad Prism 8.0.1 (GraphPad, San Diego, USA). One-way ANOVA and Student’s t-test were applied to evaluated statistical significance. *P*<0.05 was considered to indicate statistical significance.

## Results

### Characterization of the Cultured Primary MPM Cells

**Figure 1:**
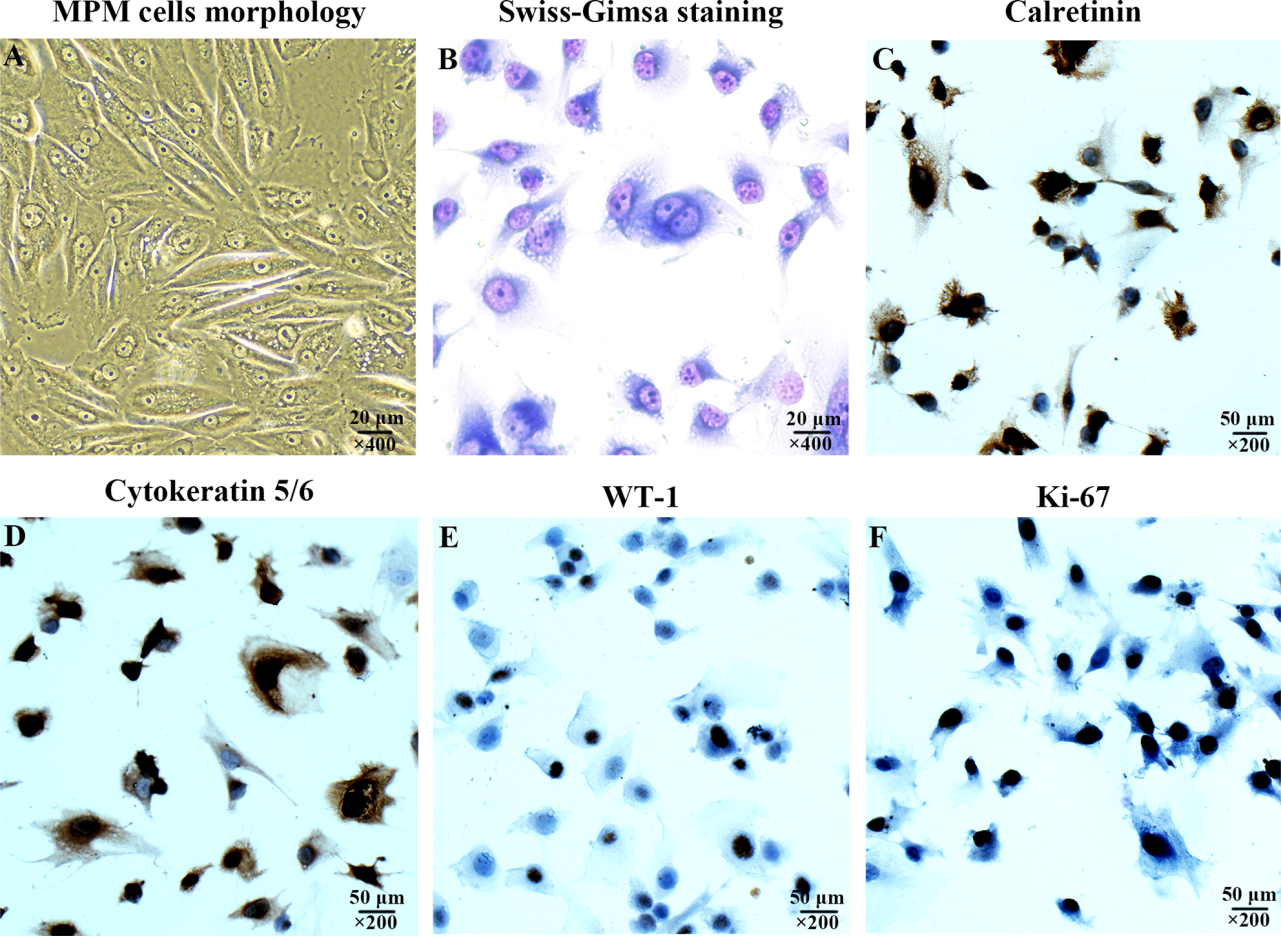
Characterization of the cultured human malignant peritoneal mesothelioma (MPM) cells. **(A)** MPM cells morphology under inverted phase contrast microscope, magnification, ×400. **(B)** MPM cell morphology was observed by Swiss-Giemsa staining, magnification, ×400. **(C)** The expression of Calretinin in MPM cells was confirmed by immunocytochemical staining, magnification, ×200. **(D)** The Cytokeratin 5/6 was positive, magnification, ×200. **(E)** The expression of WT-1 was positive, magnification, ×200. **(F)** The expression of Ki-67 was positive, magnification, ×200.

The morphology of MPM cell was observed under inverted phase contrast microscope (**Figure 1A**) and Swiss-Giemsa staining (**Figure 1B**). The cell morphology was diverse, featured by the varying size and shape, different nucleus number, and the visibility of nucleolus.

The immunocytochemical staining was performed to confirm the expression of Calretinin, Cytokeratin 5/6, WT-1, and Ki-67 were positive (**Figures 1C–F**), which were consistent with the cytological characteristics of epithelioid mesothelioma.

### Apatinib Inhibits the Viability and Proliferation of MPM Cells *In Vitro*

**Figure 2:**
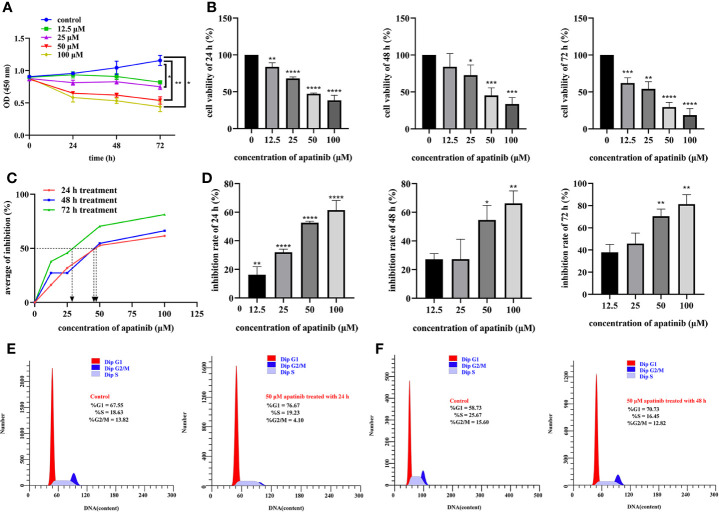
Apatinib inhibits proliferation and affects cell cycle of malignant peritoneal mesothelioma (MPM) cells. **(A)** The biological effects of apatinib on the proliferation of MPM cells (incubated with apatinib at the increasing concentrations from 0 μM to 100 μM for 24, 48, and 72 h, respectively). **(B)** The viability of MPM cells was stymied by the specified concentration of apatinib treated for 24, 48, and 72 h, respectively. The cytotoxicity of apatinib on MPM cells was confirmed by CCK-8 assays. **(C)** The IC_50_ values of apatinib in MPM cells after 24, 48, and 72 h of treatment. **(D)** The inhibition rate of apatinib on MPM cells for different time (*P < 0.05; **P < 0.01; ***P < 0.001; ****P < 0.0001). **(E, F)** Cell cycles in MPM cells were determined by flow cytometry after apatinib treatment for 24 h and 48 h, respectively.

Cells were treated with different concentrations of apatinib (0, 12.5, 25, 50 and 100 μM) for 24, 48, and 72 h, respectively. All these gradient concentrations of apatinib inhibited cells proliferation; the higher dose and longer treatment time resulted on more pronounced inhibitory effects (*P*<0.05, **Figure 2A**). In other words, the inhibitory effect of apatinib on MPM cells showed the dose-dependent and time-dependent manner. Apatinib also undermined cell viability (*P*<0.05, **Figure 2B**); IC_50_ values were shown in **Table 1** and **Figure 2C**. The inhibition rates were shown in **Figure 2D**, and the inhibitory effect was dose- dependent.

**Table 1:** IC50 values of apatinib in MPM cells for treatment time of 24 h, 48 h, and 72 h.

IC50 (μM)			
	**24 h**	**48 h**	**72 h**
MPM cells	46.34	45.14	28.73

MPM, malignant peritoneal mesothelioma.

### Apatinib Affects Cell Cycle of MPM Cells

Proportion of cells at different phases of cell cycles were compared. As shown in **Figures 2E, F**, after treatment for 24 h, for Control vs. Apatinib groups, the proportions of G1/G0 phase cells were 67.55% vs. 76.67%, of S phase cells 18.63% vs. 19.23%, and of G2/M phase cells 13.82% vs. 4.10%. After treatment for 48 h, for Control vs. Apatinib groups, the proportions of G1/G0 phase cells were 58.73% vs. 70.73%, of S phase cells 25.67% vs. 16.45%, and of G2/M phase cells 15.60% vs. 12.82%. These results indicated that apatinib induced G2/M cell cycle arrest.

### Apatinib Inhibits the Migration of MPM Cells *In Vitro*

#### Scratch Wound Healing Assay

**Figure 3:**
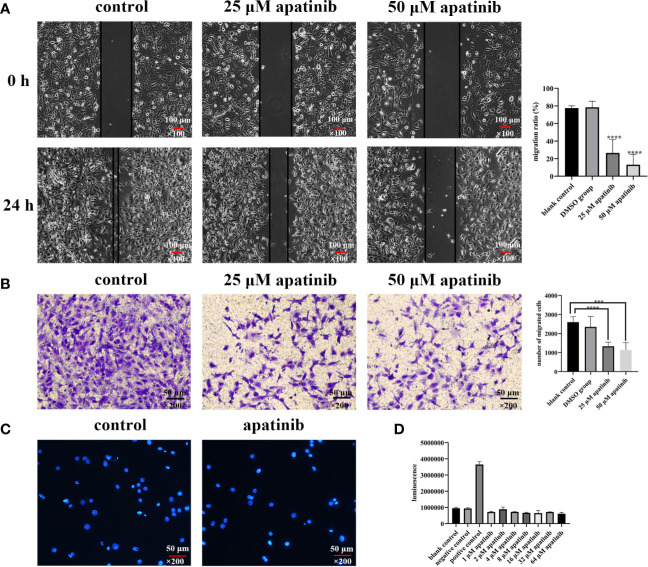
Apatinib inhibits the migration but does not induce apoptosis of malignant peritoneal mesothelioma (MPM) cells *in vitro*. **(A)** Scratch wound healing assays showed that apatinib significantly inhibited MPM cells migration (****P < 0.0001), magnification, ×100. **(B)** Transwell assay found that apatinib reduced the number of cells penetrating the membrane, as compared with control groups (***P < 0.001, ****P < 0.0001), magnification, ×200. **(C, D)** Apatinib did not induce excessive apoptosis of MPM cells, as confirmed by DAPI staining and Caspase-3 assays, magnification, ×200.

The cell migration rates in blank control, solvent control and apatinib group (25 μM and 50 μM) were (77.52 ± 2.54) %, (78.50 ± 6.91) %, (26.43 ± 15.27) %, and (13.03 ± 11.93) %, respectively, the differences between the 4 groups were statistically significant (*P*<0.0001 for all; *P*<0.0001, 25 μM *vs.* blank control; *P*<0.0001, 25 μM *vs.* solvent control; *P*<0.0001, 50 μM *vs.* blank control; *P*<0.0001, 50 μM *vs.* solvent control; *P* = 0.12, 25 μM *vs.* 50 μM; *P* = 0.75, blank control *vs.* solvent control) (**Figure 3A**).

#### Transwell Assay

The number of cells that penetrated the membrane in blank group, solvent group, apatinib group (25 μM and 50 μM) were 2601.4 ± 289.6, 2350.4 ± 556.2, 1335.8 ± 213.4, and 1138.2 ± 385.4, respectively, and the differences were statistically significant (*P*< 0.0001 for all; *P*<0.0001, 25 μM *vs.* blank control; *P* = 0.0052, 25 μM *vs.* solvent control; *P* = 0.0001, 50 μM *vs.* blank control; *P* = 0.0039, 50 μM *vs.* solvent control; *P* = 0.35, 25 μM *vs.* 50 μM; *P* = 0.40, blank control *vs.* solvent control) (**Figure 3B**).

### Apatinib Does Not Induce Apoptosis of MPM Cells

#### DAPI Staining

Apoptotic bodies were detected by using DAPI staining. Compared with blank control, no apoptosis features were detected in apatinib group (**Figure 3C**).

#### Caspase-3 Test

No difference in casepas-3 activity has been detected between control and apatinib groups (P> 0.05) (**Figure 3D**).

### Apatinib Inhibits Tumor Growth and Metastasis In Vivo

#### Establishment and Characterization of MPM PDX Model

##### Subcutaneous MPM Model

The volume of s.c. tumor reached 54.3 mm^3^ after 20 days of latency, the slow growth phase was from day 20 to 29, with a growth rate of 7.3 ± 0.7 mm^3^/day, and the rapid growth phase lasted from day 30 to day 57, with growth rate of 13.0 ± 0.3 mm^3^/day. Microscopically, tumor tissue from s.c. model was characterized as epithelioid mesothelioma.

##### MPM PDX Model

**Figure 4:**
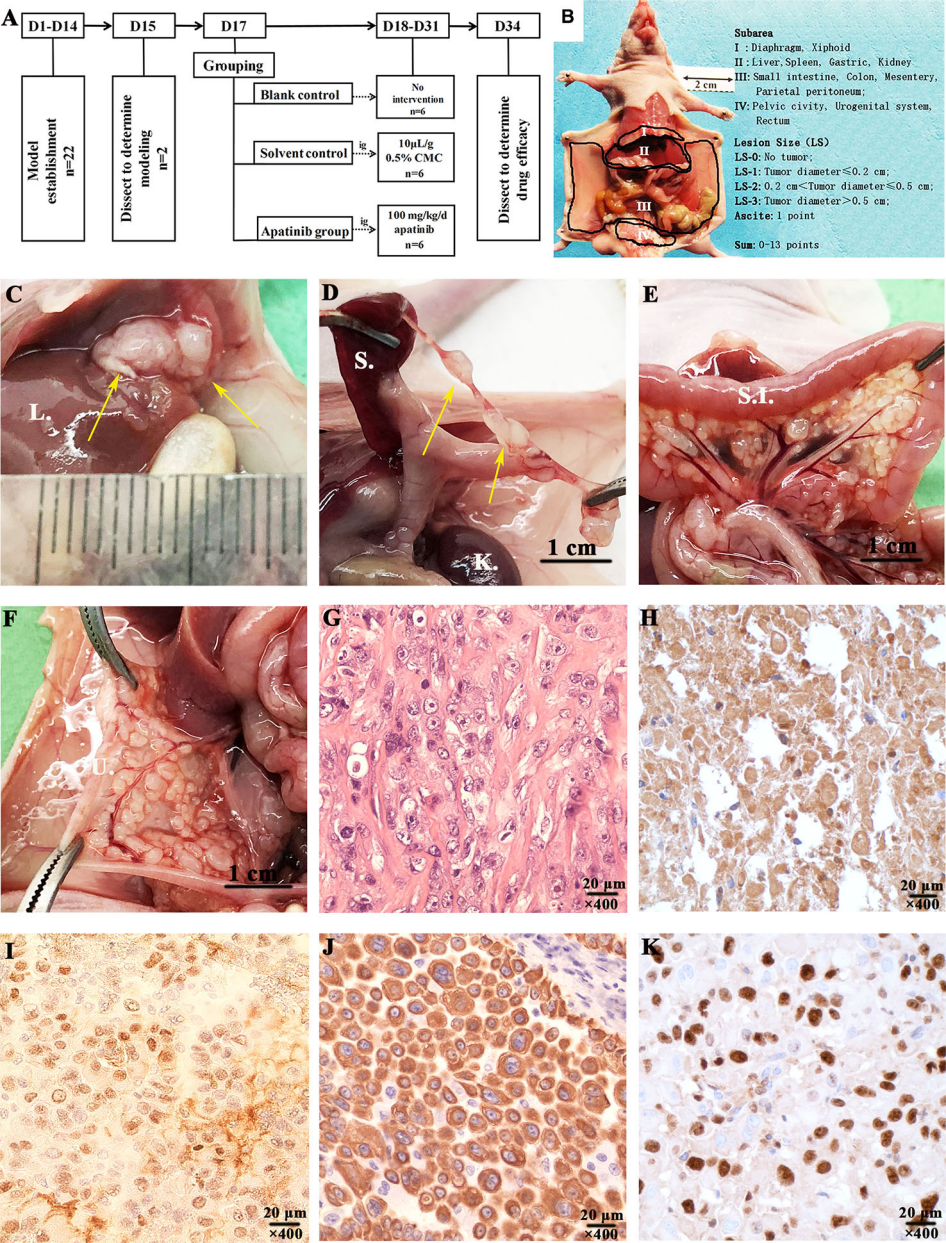
Experimental procedures and characterization of the established malignant peritoneal mesothelioma (MPM) patient-derived xenograft (PDX) models. **(A)** Experimental procedures of establishment MPM PDX models and apatinib intervention study. **(B)** The subarea and scoring of ePCI score system (16). **(C)** Tumors invaded the liver of MPM PDX models **(D)**. The mesentery of spleen was invaded by tumors. **(E)** Tumors invaded mesentery of nude mice. **(F)** The mesentery of uterus was invaded by tumors. **(G–K)** Characterization of MPM PDX models was performed by HE and immunohistochemical staining, the expressions of Calretinin, WT-1, Cytokeratin 5/6, and Ki-67 were all positive, magnification, ×400. L., liver; S., spleen; K., kidney; S.I., small intestine; U. uterus.

**Figure 5:**
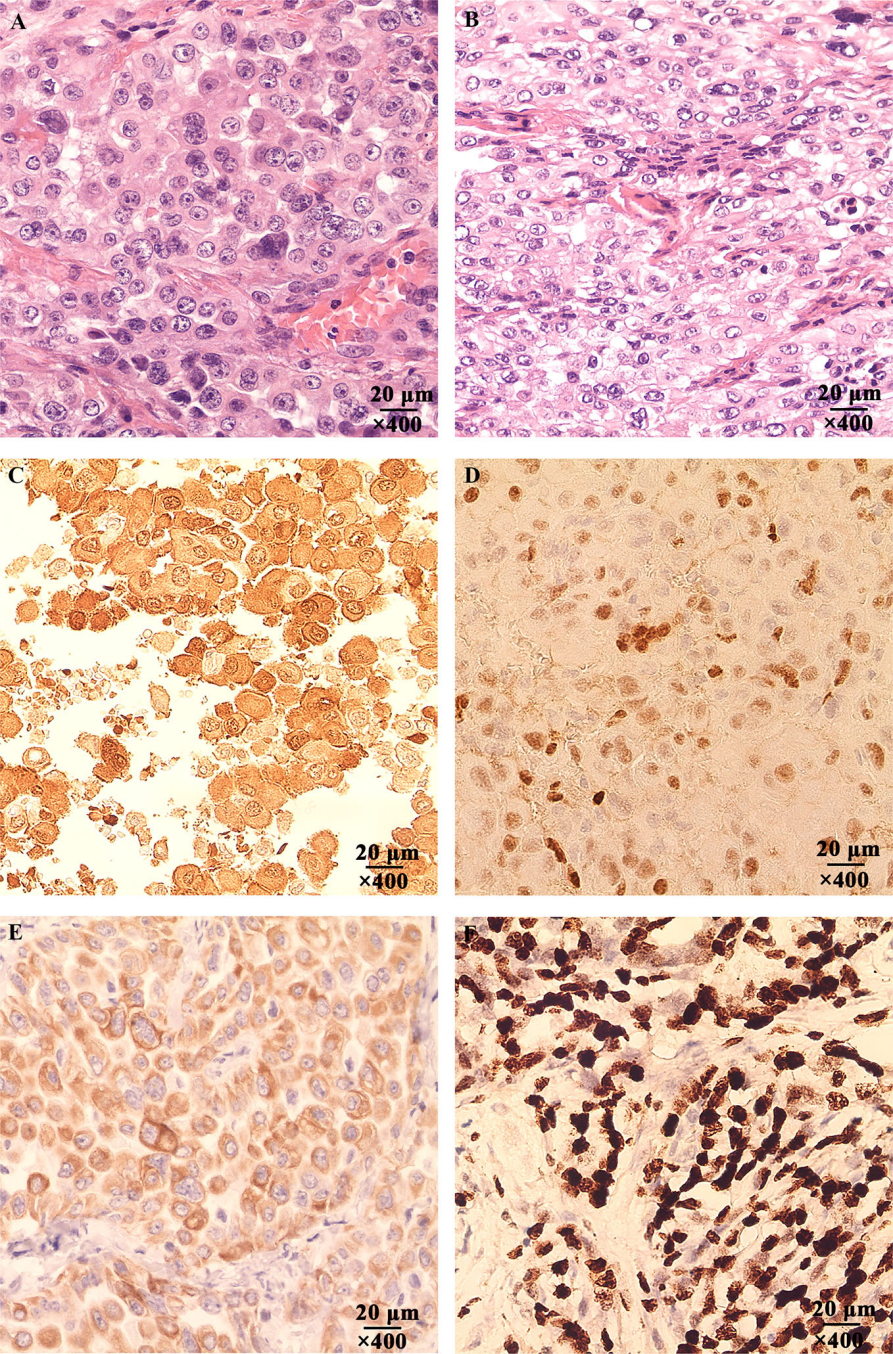
The Hematoxylin-eosin (HE) and immunohistochemical staining of the patient’s tumor tissue. **(A)** Tumor tissue of the patient invaded the stomach, magnification, ×400. **(B)** The mesentery was invaded by the tumor cells, magnification, ×400. **(C)** The expression of Calretinin was positive, magnification, ×400. **(D)** The expression of WT-1 was positive, magnification, ×400. **(E)** The expression of Cytokeratin 5/6 was positive, magnification, ×400. **(F)** The expression of Ki-67 was positive, magnification, ×400.

The flow chart of establishment models and apatinib intervention experiment are shown in **Figure 4A**. The subareas of abdominal-pelvic cavity and LS in the nude mouse model are presented in **Figure 4B**. 2 nude mice were dissected to examine the model establishment on the 15th day after inoculation. Multiple tumor invasion sites were spotted, such as diaphragm, liver, spleen, kidney, mesentery, and pelvic cavity (**Figures 4C–F**). All tumor tissues were collected for HE and immunohistochemical staining (**Figures 4G–K**). The expression of Calretinin, WT-1, Cytokeratin 5/6, and Ki-67 in models were positive, consistent with the results of the patient (**Figures 5A–F**).

#### Efficacy and Toxicity of Apatinib on MPM PDX Model

##### General Status of MPM Models

**Figure 6:**
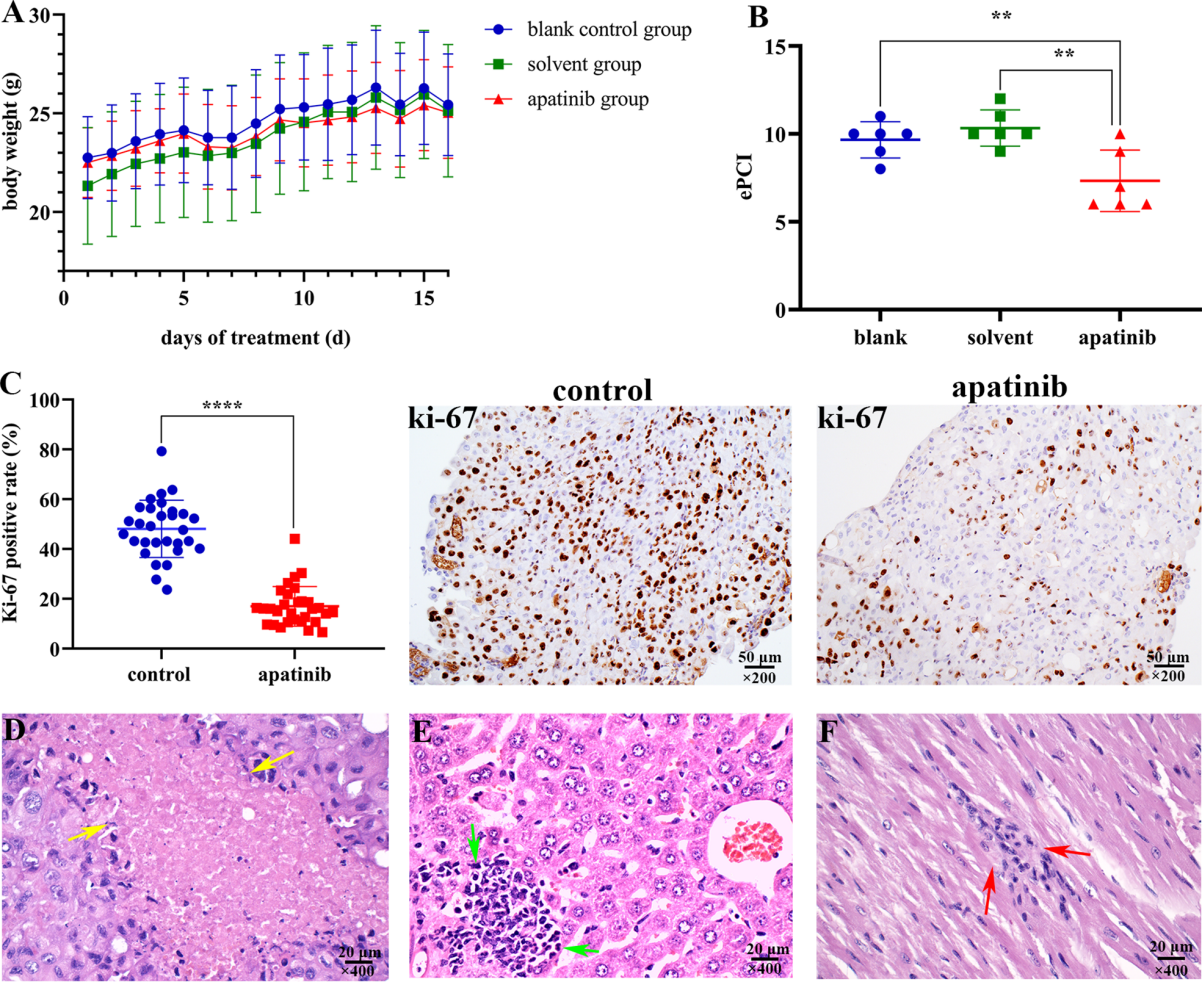
Apatinib inhibits tumor growth and metastasis *in vivo*. **(A)** Apatinib did not affect the weight of models during the treatment period. **(B)** Comparison of ePCI score among three groups, apatinib significantly decreased the ePCI compared with control groups (**P < 0.01). **(C)** The positive rate of Ki-67 in apatinib group was significantly lower than that in control group (****P < 0.0001), magnification, ×200. **(D)** Coagulation necrosis was found in apatinib treatment group, magnification, ×400. **(E)** Focal lymphoid infiltrations in the liver of apatinib group, magnification, ×400. **(F)** Focal lymphoid infiltrations in the cardiac muscle of apatinib group, magnification, ×400. ePCI, experimental peritoneal cancer index.

Apatinib did not affect the weight of nude mice, and no adverse effects were observed during the construction of mice models (**Figure 6A**).

##### Apatinib Decreases ePCI Score and the Positive Rate of Ki-67

Models were dissected to determine the extent of tumor invasion (**Table 2**). Subphrenic and liver tumor invasion were only determined in the control group.

**Table 2:** The extent of tumor invasion in three groups.

Organs	Blank control (n=6)	Solvent control (n=6)	Apatinib (n=6)
subphrenic	2/6 (33.3%)	1/6 (16.7%)	0/6 (0%)
liver	2/6 (33.3%)	2/6 (33.3%)	0/6 (0%)
pancreas	6/6 (100%)	4/6 (66.7%)	3/6 (50%)
spleen	3/6 (50%)	0/6 (0%)	1/6 (16.7%)
gastric mesentery	0/6 (0%)	1/6 (16.7%)	1/6 (16.7%)
kidney mesentery	2/6 (33.3%)	1/6 (16.7%)	1/6 (16.7%)
mesentery	5/6 (83.3%)	5/6 (83.3%)	5/6 (83.3%)
reproductive organ	5/6 (83.3%)	2/6 (33.3%)	3/6 (50%)

The comparison results of ePCI score among three groups were shown in **Figure 6B**. The ePCI score of blank control, solvent control and apatinib groups were 9.8 ± 0.9, 10.3 ± 0.9, and 7.3 ± 1.6, respectively. The differences were statistically significant (*P* = 0.003 for all; *P* = 0.008, blank control *vs.* apatinib group; *P* = 0.001, solvent control *vs.* apatinib group; *P* = 0.394, blank control *vs.* solvent control).

The positive rate of Ki-67 in apatinib group was (17.0 ± 8.0) %, which is significantly lower than that in control group (48.1 ± 11.5) % (*P* = 0.000) (**Figure 6C**); the positive rate of VEGFR-2 in apatinib group was slightly lower than that in control group, and hence indicated no statistical significance (61.4 ± 8.7% vs. 65.3 ± 9.9%; *P* = 0.119).

##### Histopathological Changes of Tumor Tissue After Apatinib Treatment

Lymphocytes infiltration, coagulation necrosis (**Figure 6D**) and eosinophilic cell fragments were found in tumor tissue of apatinib group.

##### Organ Toxicity

Focal lymphoid infiltration was detected in liver (16.7%; **Figure 6E**) and cardiac muscle (16.7%; **Figure 6F**) of apatinib group, however no pathologic changes were observed in other important organs.

## Discussion

By using surgical specimens collected from MPM patient, we established subcutaneous tumor model, PDX models and primary cell lines for *in vivo* and *in vitro* studies of the pathological mechanism and the clinical intervention for MPM. Astoul et al. previously used fresh human malignant pleural mesothelioma tissue to establish an orthotopic implantation models in nude mice, which mimicked the clinical pathological characteristics of the patient (19). However, it is not clear whether malignant mesothelioma originated from different sites, such as pleural mesothelioma and MPM, have the same genetic background (20–22). Therefore, it is indispensable to establish MPM PDX models.

In our study, we established the MPM PDX models and conducted the histopathological analysis. The HE staining and immunohistochemical results showed that the tumor tissues of the patient and the models are both epithelioid mesothelioma. However, the expression of Calretinin, WT-1 and Ki-67 in tumor tissue of the patient were stronger than that in the models. This may be caused by the following reasons: 1) Tumor heterogeneity occurred during the passage in nude mice, 2) Tumor cells from different sites had distinct proliferation ability, thus the expression of Ki-67 was different.

Many studies have reported the anti-cancer activity of apatinib in numerous cancer cells (10–14). The proliferation ability of human colorectal cancer (CRC) cell lines HT29 and HCT116 was inhibited by apatinib at different concentrations, and apatinib could induce apoptosis in these CRC cells (23). Evidence showed that apatinib significantly inhibited cell viability and increased apoptosis in both B and T lineage ALL cell lines, suppressed ALL growth and progression in a xenograft model (14). In addition, apatinib inhibited the migration of ovarian cancer cells SKOV3 and HO8910 (13). Moreover, in a case reported by Du et al. (15), a patient with plural mesothelioma received salvage treatment with apatinib alone at the dose of 500 mg/day, because of the failure of pemetrexed/cisplatin first-line chemotherapy and gemcitabine/cisplatin second-line chemotherapy. The patient had a 5-month progression-free survival. Although this case suggests that apatinib has the potential ability to treat epithelioid mesothelioma, relevant preclinical studies and clinical trials are still needed.

Recent researches also investigated molecular mechanism of apatinib in various cancer cells. On one hand, apatinib selectively competes for the ATP-binding site of VEGFR-2, blocks VEGFR-2 phosphorylation, and inhibits tumor neovascularization by suppressing downstream signaling pathways, including RAF/MEK/ERK pathway, p38-mitogen-activated protein kinase (MAPK) pathway, and phosphatidylinositol-3-kinase (PI3K)/AKT/mammalian target of rapamycin (mTOR) signaling pathway (24, 25). On the other hand, apatinib directly inhibits cancer cells activity. Cheng et al. showed that apatinib induced autophagy of CRC cells through potent stimulation of endoplasmic reticulum stress by activating the IRE1 signaling pathway (23). Apatinib also suppresses the proliferation, metastasis and induces apoptosis in many cancer cells, such as ovarian cancer cells, gastric cancer cells and cholangiocarcinoma cells, by inhibiting PI3K/AKT signaling pathways (13, 26, 27). However, the molecular mechanism underlying the effects of apatinib on MPM remains unclear. The PI3K/AKT pathway has been reported to be aberrantly activated in MPM, which plays an important role in regulating cell proliferation and survival of cancer cells (28, 29). The PI3K inhibitor and AKT-inhibitors are effective on MPM (30, 31). Moreover, the PI3K/AKT signaling pathway also induces the epithelial-mesenchymal transition (EMT) directly or through cooperation with other signaling pathways, which is critical in cancer cells invasion and metastasis (32). Based on these observations, it is likely that apatinib inhibits the proliferation and metastasis of MPM cells by inhibiting the PI3K/AKT signaling pathway and EMT. However, the study of PI3K/AKT inhibitors for malignant peritoneal mesothelioma is rare.

Thus, we applied the established models to study the biological effect of apatinib on MPM. Our results indicate that apatinib significantly inhibits MPM cells viability, proliferation, and migration, and induces cells cycle arrest at G2/M phase. Results *in vivo* show that apatinib reduces tumor burden in abdominal cavity, lowers down ePCI score, and inhibits tumor dissemination. Histopathological study reveals that apatinib results in lymphocytes infiltration, coagulation necrosis, eosinophilic cell fragments and decreased Ki-67 positive rate, without obvious histopathological toxicity. These results jointly provide evidence to use apatinib in the clinical treatment of MPM.

Although the findings from this study could have interesting clinical implications, the limitations of this study must be admitted. The most prominent limitation is the fact that this study used just one PDX model of mesothelioma. Although the tumor from the mouse model is morphologically the same as that of the patient’s tumor, it is only one histological type of mesothelioma, the epithelioid mesothelioma. Therefore, the findings and conclusions from this study should be explained with caution. These findings just suggest that under the study conditions, apatinib did have biologically meaningful inhibitory effects on epithelioid peritoneal mesothelioma. The results should not be generalized to other histological types of peritoneal mesothelioma. We had to be very clear on this point. Of course, this is only one study focusing on epithelioid peritoneal mesothelioma, and other histological types of peritoneal mesothelioma are beyond the scope of this study.

The second limitation of this study is the endpoint of the animal study. We did not use survival as the absolute endpoint in animal study, mainly because we did observe statistically significant reduction in peritoneal cancer index (PCI), a well-recognized indicator of tumor burden (16–18). Further extending the observation time was not in keeping with animal welfare.

In conclusion, despite these limitations, this study does suggest the therapeutic value of apatinib for epithelioid mesothelioma. Further clinical studies are warranted to evaluate the efficacy of apatinib in patients with epithelioid MPM and explore the molecular mechanisms.

## Data Availability Statement

The raw data supporting the conclusions of this article will be made available by the authors, without undue reservation.

## Ethics Statement

Written informed consent was obtained from the individual for the publication of any potentially identifiable images or data included in this article. All experiments were performed under the guideline of the Declaration of Helsinki and Institutional Animal Care and Use Committee Health guidelines (IACUC20190306). And the animal study was reviewed and approved by the Scientific Research Ethics Committee of Beijing Shijitan Hospital, Capital Medical University. The studies involving human participants were reviewed and approved by the Scientific Research Ethics Committee of Beijing Shijitan Hospital, Capital Medical University [Approval number: 2020 Research Ethics Review No (2).]. The participants provided their written informed consent to participate in the study.

## Author Contributions

ZR-Y was involved in study design, research implementation and data interpretation, and manuscript writing. Z-GC was involved in conduct and reporting of the work described in the article. X-MD and YL contributed to study design, data analysis and interpretation, and design of figures. All authors contributed to the article and approved the submitted version.

## Funding

This study is funded by the General Program of National Natural Science Foundation of China (No. 82073376); Beijing Municipal Administration of Hospitals' Ascent Plan (No. DFL20180701) and Beijing Municipal Grant for Medical Talents Group on Peritoneal Surface Oncology (No. 2017400003235J007).

## Conflict of Interest

Z-GC was employed by the company Thorgene Co., Ltd (Beijing, China).

The remaining authors declare that the research was conducted in the absence of any commercial or financial relationships that could be construed as a potential conflict of interest.
